# Impact of process conditions on the microbial community dynamics and metabolite production kinetics of teff sourdough fermentations under bakery and laboratory conditions

**DOI:** 10.1002/fsn3.690

**Published:** 2018-06-14

**Authors:** Henning Harth, Simon Van Kerrebroeck, Luc De Vuyst

**Affiliations:** ^1^ Research Group of Industrial Microbiology and Food Biotechnology Faculty of Sciences and Bioengineering Sciences Vrije Universiteit Brussel Brussels Belgium

**Keywords:** lactic acid bacteria, *Lactobacillus sanfranciscensis*, sourdough fermentation, teff, yeast

## Abstract

Teff and teff sourdoughs are promising ingredients for bread production. Therefore, this study aimed at the characterization of spontaneous and flour‐native starter culture‐initiated teff sourdough productions under bakery and laboratory conditions. Backslopped laboratory and bakery teff sourdough productions were characterized by different lactic acid bacteria (LAB) and yeast species, but were both characterized by a pH below 4.0 after five backslopping steps. The sourdough‐associated *Lactobacillus sanfranciscensis* was isolated for the first time from backslopped spontaneous teff sourdoughs. The autochthonous strain *L. sanfranciscensis* IMDO 150101 was tested as starter culture during laboratory teff sourdough fermentations. Its prevalence could be related to the process conditions applied, in particular the ambient temperature (below 30°C). Breads made with 20% teff sourdough (on flour basis) displayed interesting features compared with all‐wheat‐based reference breads. Teff sourdoughs were characterized as to their pH evolution, microbial community dynamics, and microbial species composition. Representative strains of the LAB species isolated from these sourdoughs, in particular *L. sanfranciscensis*, may be selected as starter cultures for the production of stable teff sourdoughs and flavorful breads, provided they are adapted to the environmental conditions applied.

## 1 INTRODUCTION

Fermentations of flour‐water mixtures, resulting in a sourdough, are carried out both spontaneously and starter culture‐initiated (De Vuyst, Van Kerrebroeck, & Leroy, 2017; De Vuyst, Vrancken, Ravyts, Rimaux, & Weckx, 2009; De Vuyst et al., 2014; Minervini, De Angelis, Di Cagno, & Gobbetti, 2014; Minervini et al., 2010; Siragusa et al., 2009; Van Kerrebroeck, Maes, & De Vuyst, 2017). Concerning the former, often backslopping is applied. Lactic acid bacteria (LAB) and yeasts growing in the cereal matrix originate from the flour itself, other dough ingredients, or the environment (Minervini et al., 2010; Scheirlinck, Van der Meulen, De Vuyst, Vandamme, & Huys, 2009; Scheirlinck et al., 2007; Siragusa et al., 2009). Sourdough‐based baked goods are produced and consumed worldwide because of their natural and artisan character, technological advantages, nutritional properties, and health‐promoting effects (Arendt, Ryan, & Dal Bello, 2007; Gobbetti, Rizzello, Di Cagno, & De Angelis, 2014; Katina et al., 2005; Poutanen, Flander, & Katina, 2009).

Backslopped laboratory sourdoughs based on spontaneous wheat, rye, spelt, and barley fermentations, with the flour as the sole nonsterile component, have been studied in detail (Harth, Van Kerrebroeck, & De Vuyst, 2016; Van der Meulen et al., 2007; Vrancken, Rimaux, Weckx, Leroy, & De Vuyst, 2011; Weckx, Van der Meulen, Allemeersch et al., 2010; Weckx, Van der Meulen, Maes et al., 2010; Weckx et al., 2011). The prevalence of *Lactobacillus fermentum* (strictly heterofermentative), *Lactobacillus plantarum* (facultatively heterofermentative), and/or *Leuconostoc citreum* (strictly heterofermentative) in wheat, rye, and spelt sourdoughs depends on the incubation temperature and backslopping time. Laboratory barley sourdoughs harbor *Lactobacillus brevis* too (Harth et al., 2016). These LAB species mainly produce lactic acid (homo‐ and heterofermenters) and acetic acid (heterofermenters). The yeasts *Candida glabrata* and *Wickerhamomyces anomalus* have commonly been found in laboratory wheat sourdoughs, whereas *Saccharomyces cerevisiae* has commonly been found in laboratory barley sourdoughs (Harth et al., 2016; Vrancken et al., 2010).

Teff (*Eragrostis tef*) is a tropical cereal (C_4_‐plant belonging to the family *Poaceae*), probably originating from Ethiopia and being one of the earliest plants domesticated (Arendt & Zannini, 2013; Ashenafi, 2006; Gebremariam, Zarnkow, & Becker, 2014). In Ethiopia, teff flour is subjected to a traditional two‐step fermentation process lasting for about 24–72 hr, depending on the ambient temperature, and making use of a backslopping procedure (*ersho*). The sourdough obtained is processed into flat breads (*injera*). Also, porridge and beer (*tella*) are made from teff. Today, teff is cultivated in Ethiopia (staple food; 25% of its total cereal crop production), South‐Africa (forage crop), the United States (health grain), and The Netherlands (health grain) (Miller, 2010; Tefera & Belay, 2006; Vos, van Delden, & Stomph, 2013).

Whereas different parts of the kernel can be fractionated in the case of wheat, rye, and barley, the whole kernel is used in the case of teff because of its small size. This may influence the fermentation process because of the presence of bran (Katina, Liukkonen et al., 2007; Katina, Laitila et al., 2007; Prückler et al., 2015). Teff contains many proteins (providing all essential amino acids, including lysine), slowly digestible complex carbohydrates (causing satiety), many fibers (improving gut health), and more bioavailable minerals (among which calcium and iron) (Ashenafi, 2006; Gebremariam et al., 2014). These properties make teff an interesting product for human consumption in general and for consumption by elderly, veganists, and sport men in particular. Given its high‐quality protein and lacking gluten, it is very promising for the production of gluten‐free breads (Campo, del Arco, Urtasun, Oria, & Ferrer‐Mairar, 2016; Gebremariam et al., 2014; Moroni, Arendt, & Dal Bello, 2011; Moroni, Arendt, Morrissey, & Dal Bello, 2010; Moroni, Dal Bello, & Arendt, 2009; Wolter et al., 2014).

Early microbiological studies of teff flour and fermenting dough have been reviewed (Ashenafi, 2006). The predominance of *L. brevis*,* L. fermentum*,* L. plantarum*, and/or *Pediococcus pentosaceus* has been shown by means of phenotypic tests (Desiye & Abegaz, 2013; Gashe, 1985) and randomly amplified polymorphic DNA profiling (Nigatu, 2000). *Candida*,* Saccharomyces*, and/or *Torulopsis* are the dominating yeast genera (Desiye & Abegaz, 2013; Gifawesen & Besrat, 1982). Using both culture‐dependent and culture‐independent techniques, stable teff sourdoughs are dominated by several *Lactobacillus* spp. and *C. glabrata*,* Kazachstania barnettii*, or *S. cerevisiae*, depending on the dough yield, incubation temperature, backslopping time, and/or mixed‐strain starter culture used (Moroni et al., 2010, 2011). The prevalence of the LAB species *L. brevis* (Moroni et al., 2010) and *Lactobacillus pontis* (Moroni et al., 2011) has indicated their competitiveness during teff sourdough fermentation. Also, *Lactobacillus sanfranciscensis* strains survive teff sourdoughs (Moroni et al., 2010), indicating that this LAB species is not dedicated to wheat, rye, and spelt sourdoughs solely (De Vuyst et al., 2014; Hammes et al., 2005).

The aim of this study was to determine the LAB and yeast species diversity, microbial community dynamics, and metabolite production kinetics of spontaneous teff sourdough fermentations performed through backslopping under bakery and laboratory conditions and to assess the competitiveness of *L. sanfranciscensis* IMDO 150101 as starter culture strain for teff sourdoughs, to be able to develop stable teff sourdoughs for bread production.

## 2 MATERIALS AND METHODS

### 2.1 Flour

Three batches of teff flour (A, B, and C) were used throughout this study. They were provided by Prograin International (batches A and B; Hooghalen, The Netherlands) and a local bakery (batch C; Limburg, Belgium). They contained (m/m) 12.0%, 11.2%, and 10.0% moisture; 55.3%, 75.9%, and 72.0% carbohydrates; 11.7%, 8.8%, and 10.0% proteins; 1.8%, 1.5%, and 2.0% fat; and 1.8%, 1.2%, and 1.9% ash, respectively.

### 2.2 Sourdough productions

#### 2.2.1 Spontaneous backslopped sourdough productions

Spontaneous teff sourdough fermentations were carried out through backslopping, both in a small industrial bakery [in open vessels, 8 kg, low dough yield of 200, refreshment every 24 hr during 10 days, room temperature (the temperature averaged 23°C after refreshment and decreased to an average of 18°C after 24 hr of incubation due to the cool temperature at night), in biological triplicate due to the inconsistent conditions] and in the laboratory (in fermentors, 8 L, high dough yield of 400, refreshment every 24 hr during 10 days, controlled temperature of 30°C, in biological duplicate), as described previously, whereby teff flour was used instead of barley flour (Harth et al., 2016). The bakery sourdough productions are referred to as TF1′ (flour A), TF2′ (flour A), and TF3′ (flour B), and the laboratory sourdough productions are referred to as TF1 (flour A) and TF2 (flour B).

#### 2.2.2 Starter culture‐initiated sourdough productions

Starter culture‐initiated laboratory sourdough fermentations were carried out with the *L. sanfranciscensis* IMDO 150101 strain, which was isolated from backslopped teff sourdough production TF1′ during this study. Both small‐scale fermentations in glass bottles (350 mL, dough yield of 400) and fermentor‐scale fermentations (8 L, dough yield of 400) were carried out. The former were performed to assess the impact of the temperature on the survival and prevalence of the *L. sanfranciscensis* strain used, whereas the latter allowed a comparison of teff fermentations with laboratory nonteff sourdough fermentations carried out before (Van der Meulen et al., 2007; Van Kerrebroeck, Bastos, Harth, & De Vuyst, 2016; Vrancken et al., 2011; Weckx, Van der Meulen, Allemeersch et al., 2010; Weckx, Van der Meulen, Maes et al., 2010). The flour‐water mixtures were inoculated with a cell suspension of the starter culture strain at a final concentration of 10^6^–10^7^ colony forming units (CFUs)/mL. The glass bottles were shaken at 160 revolutions per minute (rpm) to prevent sedimentation of flour particles and were incubated at 23°C, 30°C, or 37°C for 120 hr. The temperature of the fermentors was kept constant at 30°C for 72 hr; the mixture was kept homogeneous through stirring at 300 rpm. All starter culture‐initiated fermentations were performed in triplicate. They are further referred to as TFSC23, TFSC30, and TFSC37 (flour B, small‐scale fermentations) and as TFFS1, TFFS2, and TFFS3 (flour C, fermentor‐scale fermentations).

### 2.3 Sampling and analyses

Sampling procedure, determination of pH and total titratable acidity (TTA), culture‐dependent (plating on different agar media, from which colonies were picked up) and culture‐independent microbial community dynamics [denaturing gradient gel electrophoresis of targeted PCR amplicons from sample DNA, PCR‐DGGE; LAB, all sourdoughs (and flours); yeasts; all sourdoughs (and flours), except for the small‐scale fermentations), classification and identification of LAB [all sourdoughs (and flours); (GTG)_5_‐PCR fingerprinting] and yeast isolates [backslopped sourdoughs (and flours); M13‐PCR fingerprinting], and metabolite target analyses for all sourdough productions were carried out as described previously (Harth et al., 2016).

#### 2.3.1 Culture‐dependent analysis

The agar media used were de Man‐Rogosa‐Sharpe‐5 (mMRS‐5) agar medium (Meroth, Walter, Hertel, Brandt, & Hammes, 2003), supplemented with cycloheximide (final concentration of 0.1 g/L; Sigma‐Aldrich, Saint Louis, MO, USA) for LAB isolation (including the *L. sanfranciscensis* IMDO 150101 strain) and yeast extract‐peptone‐glucose (YPG) agar medium (Meroth, Hammes, & Hertel, 2003), chloramphenicol being present in a final concentration of 0.1 g/L, for yeast isolation. Incubations were performed at 30°C for 48 to 72 hr. Also, samples from the flour batches were analyzed microbiologically. Therefore, 10 g of flour was mixed with 10 mL of saline (0.85% NaCl, m/v), a tenfold dilution series of these suspensions was made, and 100 μL of each dilution was plated on mMRS‐5 and YPG agar media supplemented with cycloheximide or chloramphenicol in a final concentration of 0.1 g/L, respectively.

#### 2.3.2 Culture‐independent analysis

The primers used for 16S rRNA‐PCR‐DGGE bacterial community profiling were the bacterial universal primers F357‐518R (Øvreås, Forney, Daae, & Torsvik, 1997); those for 26S rRNA‐PCR‐DGGE fungal community profiling were the eukaryotic universal primers NL1‐LS2 (Cocolin, Bisson, & Mills, 2000). The conditions applied were as described previously (Harth et al., 2016).

#### 2.3.3 Metabolite target analysis

The concentrations of glucose, fructose, sucrose, maltose, and mannitol were determined by high‐performance anion exchange chromatography with pulsed amperometric detection, those of ethanol and acetic acid by gas chromatography with flame ionization detection, and those of lactic acid by high‐performance liquid chromatography with refractive index detection, as described previously (Harth et al., 2016). Volatile compounds were determined qualitatively through gas chromatography coupled to mass spectrometry in conjunction with solid‐phase microextraction of the sourdough headspace (HS/SPME‐GC‐MS), as described previously (Harth et al., 2016).

### 2.4 Statistical analysis of volatile compound data

A principle component analysis (PCA) was performed on the peak area data of the HS/SPME‐GC‐MS volatile analysis. This was followed by a cluster analysis, using the software package SPSS 20.0 (SPSS, Chicago, IL, USA). To determine the number of principal components (PCs), a scree plot was constructed. To maximize the sum of the squares of the correlations between the original variables and the rotated PCs (factor loadings), a Varimax with Kaiser normalization rotation was applied. A three‐dimensional score plot was constructed.

### 2.5 Bread production and evaluation

Teff sourdough‐based breads were produced in the pilot plants of four industrial bakeries according to their respective recipes and breadmaking conditions, with addition of 20% (m/m, on flour basis) teff sourdough from the backslopped (after backslopping step 10) sourdough productions (bakery sourdough productions TF1′, TF2′, and TF3′, and laboratory sourdough productions TF1 and TF2), and from the starter culture‐initiated sourdough production TFFS1. In addition to, all‐wheat‐based reference breads (without the addition of teff sourdough) were produced. Parbaked breads were produced at the last fermentation day and baked off prior to evaluation. The breads were assessed by 21 consumers on the basis of descriptive data.

## 3 RESULTS

### 3.1 pH and TTA evolution

#### 3.1.1 Backslopped bakery sourdough productions

**Figure 1:**
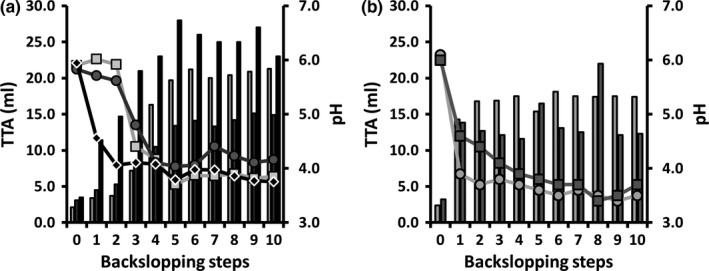
Evolution of pH (lines) and total titratable acidity (TTA; bars) during teff sourdough productions carried out under bakery conditions (a; TF1′, light gray; TF2′, dark gray; TF3′, black) and laboratory conditions (b; TF1, light gray; TF2, dark gray)

During the first two backslopping steps of the spontaneous backslopped bakery teff sourdough productions TF1′and TF2′, no significant change in pH and TTA occurred, whereas the pH dropped from 5.9 to 4.6 and the TTA increased from 3.5 to 11.4 mL for backslopped bakery teff sourdough production TF3′ (Figure 1a). After the third backslopping step, the pH of TF1′ and TF2′ dropped from 5.9 and 5.6 to 4.4 and 4.8, respectively, and further down to 3.9 and 4.1 at the end of both backslopping processes. In the case of TF3′, the pH continuously dropped to reach a final value of 3.7. Both bakery teff sourdough productions TF1′ and TF2′ showed a continuous increase of the TTA from the third backslopping step onwards. Bakery sourdough production TF3′ showed an increase of the TTA value to 28.0 mL after five backsloppings. At the end of the backslopping processes for TF1′ and TF2′, TTA values of 21.3 and 14.9 mL, respectively, were reached. The TTA of the teff sourdough production TF3′ continuously dropped from the fifth backslopping step onwards to a final value of 23.1 mL at the end of the backslopping process.

#### 3.1.2 Backslopped laboratory sourdough productions

The pH of the spontaneous backslopped laboratory teff sourdough productions TF1 and TF2 decreased from 6.1 to 3.9 and from 6.0 to 4.6 after the first 24 hr of fermentation, respectively, while the TTA values increased from 2.4 to 14.3 mL and from 3.2 to 13.8 mL, respectively. During these laboratory sourdough productions, both the pH and TTA evolved faster compared with the bakery ones (Figure 1b). After seven backsloppings, the pH reached an average value of 3.7 and remained more or less stable upon further backslopping for both processes, while the TTA values increased toward an average value of 18.1 and 13.1 mL for the respective backslopping processes. A pH of 3.5 and 3.7 was reached at the end of the respective backslopping processes, which corresponded with TTA values of 17.4 and 12.3 mL.

#### 3.1.3 Starter culture‐initiated laboratory sourdough fermentations

During the first 24 hr of all *L. sanfranciscensis* IMDO 150101‐initiated laboratory teff sourdough fermentations, the pH decreased from 6.0 to 6.2 to values between 3.6 and 4.0, with the exception of TFSC37 (average value of 4.2). After 72 hr of fermentation, average values of 3.6 (TFSC23, TFSC30, and TFFS1), 3.9 (TFFS2 and TFFS3), and 4.1 (TFSC37) were reached.

### 3.2 Culture‐dependent LAB and yeast community dynamics and identifications

#### 3.2.1 Teff flours

In total, 17, 41, and 24 isolates of the bacterial communities of teff flours A, B, and C, respectively, were identified. They belonged to five, four, and three different bacterial species, respectively. In the case of flour A, the largest cluster of isolates represented *Weissella confusa* (29%), followed by *Lactobacillus sakei* (28%), *L. sanfranciscensis* (24%), *Lactobacillus coryniformis* (11%), and *Le. citreum* (8%). In the case of flour B, the largest cluster of isolates represented *Weissella cibaria* (44%), followed by *Lactococcus lactis* (27%), *Pediococcus acidilactici* (17%), and *L. plantarum* (12%). In the case of flour C, the largest cluster of isolates represented *W. cibaria/confusa* (54%), followed by *L. fermentum* (42%), and *L. plantarum* (4%). Yeasts could not be picked up, as their counts were below the detection limit.

#### 3.2.2 Backslopped bakery sourdough productions

**Figure 2:**
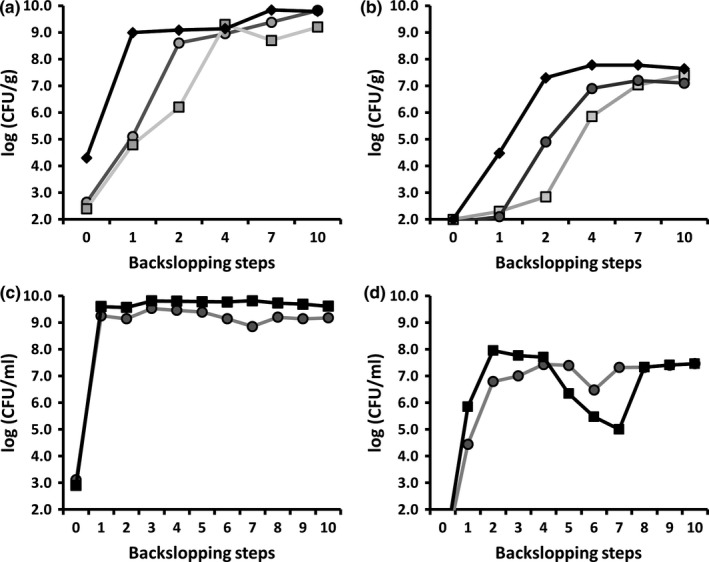
Culture‐dependent community dynamics expressed as microbial counts of the lactic acid bacteria (a,c) and yeasts (b,d) during teff sourdough productions carried out under bakery conditions (a,b; TF1′, light gray; TF2′, dark gray; TF3′, black) and laboratory conditions (c,d; TF1, light gray; TF2, dark gray)

During the backslopped bakery teff sourdough productions TF1′and TF2′, low LAB and yeast counts of <10^3^ CFU/g and <10^2^ CFU/g, respectively, were found at the start (Figure 2a,b). The initial LAB counts (10^4^ CFU/g) of backslopped bakery teff sourdough production TF3′ were higher and increased faster, whereas the yeast counts were the same but increased faster, compared with TF1′ and TF2′. The LAB counts of TF1′ and TF2′ reached values of approximately 10^9^ CFU/g after the fourth backslopping step, whereas the yeast counts reached values of approximately 10^7^ CFU/g after the seventh backslopping step and remained stable upon further backslopping. In the case of TF3′, high LAB counts of 10^9^ CFU/g were reached after the first backslopping step, which remained more or less stable until the end of the backslopping process. The yeast counts of TF3′ reached values of >10^7^ CFU/g after the second backslopping step and remained constant until the end of the backslopping process.

**Figure 3:**
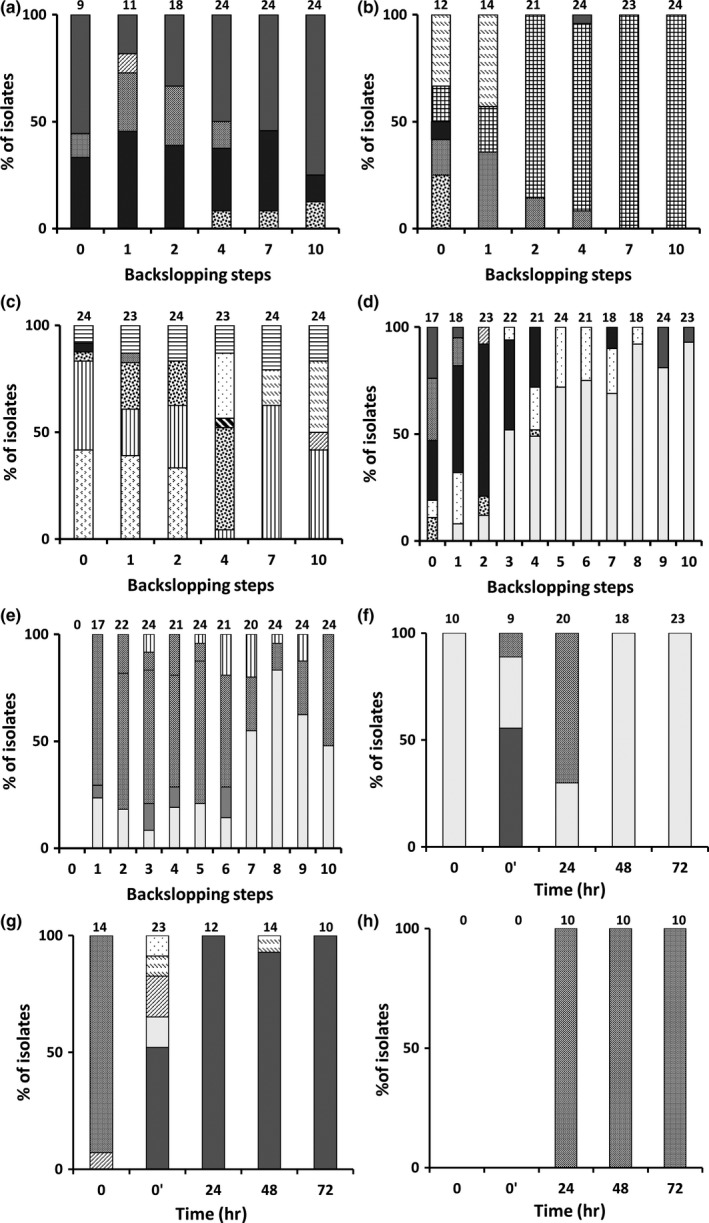
Culture‐dependent community dynamics and identifications of the lactic acid bacteria (LAB) during spontaneous teff sourdough productions carried out under bakery conditions (a, TF1′; b, TF2′; c, TF3′) and laboratory conditions (d, TF1; e, TF2) and during *Lactobacillus sanfranciscensis* IMDO 150101‐initiated fermentor‐scale laboratory teff sourdough productions (f, TFFS1; g, TFFS2; h, TFFS3). The number of isolates identified is indicated on top of the bars. The LAB species were identified as *L. sanfranciscensis* (100% identity; accession no. EU350220.1), 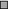; *Lactobacillus sakei* (99% identity; accession no. AB362731.1), 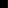; *Leuconostoc citreum* (98% identity; accession no. NR041727.1), 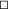; *Lactobacillus coryniformis* (99% identity; accession no. AB778519.1), 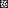; *Lactobacillus fermentum* (100% identity; accession no. LC065036.1), 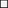; *Weissella cibaria/confusa* (99% identity; accession no. LC096236.1/LC063164.1), 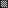; *Lactobacillus plantarum/pentosus/paraplantarum* (99% identity; accession no. LC071808.1/LC064896.1/NR_025447.1), 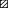; *Pediococcus pentosaceus/clausseni* (99% identity; accession no. LC071837.1/NR_075029.1), 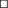; *Lactobacillus brevis* (99% identity; accession no. AB024299.1), 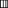; *Pediococcus acidilactici* (99% identity; accession no. NR042057.1), 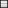; *Lactobacillus helveticus* (99% identity; accession no. FJ749441.1), 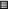; *Lactococcus lactis* (99% identity; accession no. DQ212982.1), 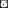; *Leuconostoc mesenteroides* (99% identity; accession no. EU259610.1), 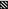; and not identified, 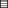

During each of the backslopped bakery teff sourdough productions, more than 100 LAB isolates were obtained from mMRS‐5 agar media, the identities of which are represented in Table 1. *Lactobacillus sanfranciscensis* was isolated from mainly TF1′ (45%). During TF1′, *L. sanfranciscensis*,* W. cibaria/confusa*, and *L. sakei* were present from the beginning of the backslopping process, with an increasing relative abundance of *L. sanfranciscensis* and a decreasing one of *L. sakei* upon backslopping (Figure 3a). *Weissella cibaria/confusa* was outcompeted after the fourth backslopping step, whereas *L. coryniformis* grew out from then on. During TF2′, *P. acidilactici* and *W. confusa* were outcompeted by *L. helveticus*, which was dominant from the second backslopping step (Figure 3b). *Lactobacillus helveticus* represented 77% of all isolates, albeit that it was not isolated from the other backslopped bakery teff sourdough productions. In the case of TF3′, *L. brevis* was isolated throughout the whole backslopping process and became the dominant LAB species at the end of the backslopping process, although accompanied with *P. pentosaceus* that was present from backslopping step 7 (Figure 3c). *Lactococcus lactis* decreased in relative abundance from the start till backslopping step 2, whereas *L. coryniformis* was present till the fourth backslopping step.

**Table 1:** Species diversity of lactic acid bacteria during bakery and laboratory teff sourdough productions: backslopped bakery sourdoughs (TF1′, TF2′, and TF3′), backslopped laboratory sourdoughs (TF1 and TF2), small‐scale *Lactobacillus sanfranciscensis* IMDO 150101‐initiated laboratory sourdoughs fermented at 23, 30, and 37°C (TFSC23, TFSC30, and TFSC37, respectively), and fermentor‐scale *L. sanfranciscensis* IMDO 150101‐initiated laboratory sourdoughs (TFFS1, TFFS2, and TFFS3)

LAB species	Occurrence in sourdoughs (%)										
	TF1′	TF2′	TF3′	TF1	TF2	TFSC23	TFSC30	TFSC37	TFFS1	TFFS2	TFFS3
*Lactobacillus brevis*			34		7						
*Lactobacillus coryniformis*	14	3	15	3							
*Lactobacillus fermentum*				55	36		11	47	74	5	
*Lactobacillus helveticus*		77									
*Lactobacillus plantarum*	3	1	1	1	4	2				8	
*Lactobacillus sakei*	33	1	1	21							
*Lactobacillus sanfranciscensis*	45	1		5		85	73	19	6	58	
*Lactococcus lactis*			19								
*Leuconostoc citreum*			5	12							
*Leuconostoc mesenteroides*			1								
*Pediococcus acidilactici*		8					5	13		5	
*Pediococcus pentosaceus*			8				5	13		4	
*Weissella cibaria/confusa*	4	10	1	3	53	13	6	8	20	20	100

Based on M13‐PCR fingerprinting, 100% of the 73, 86, and 77 colonies picked up from YPG agar media for the backslopped bakery teff sourdough productions TF1′, TF2′, and TF3′, respectively, belonged to the yeast species *Kazachstania exigua*.

#### 3.2.3 Backslopped laboratory sourdough productions

Low LAB counts of 10^3^ CFU/mL and no yeast counts were found at the start of both backslopped laboratory teff sourdough productions TF1 and TF2 (Figure 2c). These LAB counts reached values of >10^9^ CFU/mL after the first backslopping step, whereas the yeast counts reached values of ≥10^7^ CFU/mL after the second backslopping step (Figure 2c,d). Afterward, the LAB counts remained stable. However, the yeast counts were lower during the fifth, sixth, and seventh backslopping steps.

More than 200 colonies were picked up from mMRS‐5 agar media from plated samples of the backslopped laboratory teff sourdough productions TF1 and TF2, the identities of which are represented in Table 1. During the first two backslopping steps of TF1, *L. coryniformis*,* L. fermentum*,* L. sakei*,* L. sanfranciscensis*,* Le. citreum*, and *W. cibaria/confusa* were found as LAB species. The relative abundance of *L. fermentum* increased during TF1 till the end of the backslopping process, accompanied with *L. sakei* during backslopping steps 2–4, *Le. citreum* during backslopping steps 4–8, and *L. sanfranciscensis* during backslopping steps 9–10 (Figure 3d). At the beginning of TF2, *L. plantarum*,* W. cibaria/confusa,* and *L. fermentum* were found (Figure 3e). *Weissella cibaria/confusa* persisted throughout this whole backslopped sourdough production process, decreasing in abundance upon backslopping step 7. From then on, *L. fermentum* became the most prevalent LAB species. *Lactobacillus plantarum* and *L. brevis* were present sporadically during backslopping steps 1–6 and 3–9, respectively.

Based on M13‐PCR fingerprinting of genomic DNA, 100% of the 187 and 153 colonies picked up from YPG agar media for both backslopped laboratory teff sourdough productions TF1 and TF2 belonged to the yeast species *S. cerevisiae*.

#### 3.2.4 Starter culture‐initiated laboratory sourdough fermentations

Concerning the impact of the fermentation temperature on the prevalence of *L. sanfranciscensis* IMDO 150101 as added starter culture strain for teff sourdoughs, the small‐scale fermentations showed that this strain was able to prevail in the fermentations performed at 23°C and 30°C (Table 1). At 30°C, *L. sanfranciscensis* IMDO 150101 prevailed until 48 hr of fermentation. However, it was outcompeted by *L. fermentum* and *P. acidilactici* after 72 hr of fermentation. At 37°C, *L. fermentum*,* P. acidilactici*, and *P. pentosaceus* were the prevailing LAB species.

Prior to inoculation of the starter culture‐initiated fermentor‐scale teff sourdough fermentations, low initial LAB counts (3.0 log CFU/mL) and even lower initial yeast counts (<3.0 log CFU/mL) were present. After 24 hr of fermentation, stable LAB counts between 8.0 and 9.7 log CFU/mL were found, except for TFFS3, in which they declined. The added starter culture strain did not prevail during TFFS1 and TFFS3, but it did during TFFS2. Instead, the former fermentations were characterized by the prevalence of *L. fermentum* and *W. cibaria/confusa*, respectively. The latter species was retrieved either as the sole LAB species (TFFS3) or in combination with other LAB species (TFFS1 and TFFS2). After 72 hr of fermentation, yeast counts between 5.5 and 7.0 log CFU/mL were found.

### 3.3 Culture‐independent community dynamics and identifications of LAB and yeasts

#### 3.3.1 Backslopped bakery sourdough productions

**Figure 4:**
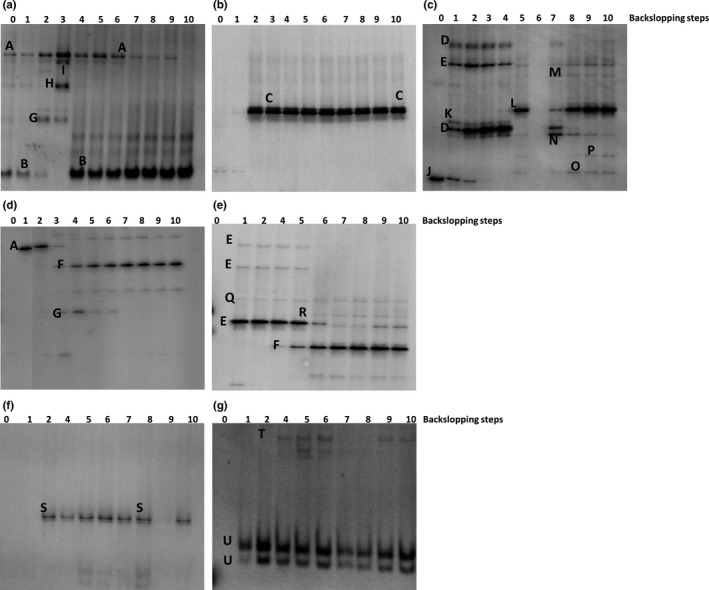
Culture‐independent bacterial (a–e) and fungal (f,g) community dynamics through 16S rRNA‐PCR‐ and 26S rRNA‐PCR‐DGGE (universal primers) analysis during teff sourdough productions carried out under bakery conditions (a, TF1′; b, TF2′; c,f, TF3′) and laboratory conditions (d, TF1; e,g, TF2). The letters accompanying the bands correspond to the following identities: A, *Lactobacillus sakei* (99% identity; accession no. AB362731.1); B, *Lactobacillus sanfranciscensis* (99% identity; accession no. EU350220.1); C, *Lactobacillus helveticus* (99% identity; accession no. FJ749441.1); D, *Lactobacillus sakei/curvatus/graminis* (98% identity; accession no. AB362731.1/NR114915.1/AJ621551.1); E, *Weissella cibaria/confusa*; (99% identity; accession no. LC063164.1/LC096236.1); F, *Lactobacillus fermentum* (99% identity; accession no. LC065036.1); G, *Leuconostoc citreum* (99% identity; accession no. NR041727.1); H, *Weissella* sp. (97% identity; accession no. AF086707.1); I, no PCR amplicon; J, plant DNA (99% identity; accession no. HM802264.1); K, *Lactobacillus sakei/fuchuensis* (98% identity; accession no. KM267630.1/AB470236.1); L, *Lactobacillus brevis* (99% identity; accession no. AB024299.1); M, *Lactobacillus plantarum/brevis* (99% identity; accession no. AB741780.1/AB024299.1); N, *Pediococcus pentosaceus* (99% identity; accession no. LC071837.1); O, *Lactobacillus manihotivorans* (99% identity; accession no. KF418821.1); P, *Lactobacillus farciminis* (99% identity; accession no. M58817.2); Q, *Lactobacillus reuteri/fermentum* (98% identity; accession no. LC097076.1/LC063167); R, *Lactobacillus reuteri/fermentum/panis* (98% identity; accession no. LC097076.1/LC063167.1/LC064899.1); S, *Kazachstania exigua* (100% identity; accession no. KC111879.1); T, *Saccharomyces cerevisiae* (99% identity; accession no. NG042623.1); and U, plant DNA (99% identity; accession no. HM802264.1)

Culture‐independent analysis based on 16S rRNA‐PCR‐DGGE bacterial community profiling revealed three different phases during the backslopped bakery teff sourdough productions TF1′ and TF3′ (Figure 4a,b). The first phase (backslopping step 1) of TF1′ was dominated by *L. sakei* and *L. sanfranciscensis*, followed by a second phase (backslopping steps 2–6) that was represented by *L. sakei* (throughout), *Le. citreum* (backslopping steps 2 and 3), and a *Weissella* sp. (backslopping step 3) (Figure 4a). The last phase of the backslopping process (backslopping steps 7–10) was dominated by *L. sanfranciscensis*. In the case of TF2′, only one phase occurred that was dominated by *L. helveticus* (Figure 4b). The first phase of TF3′ (backslopping steps 1–4) was represented by *L. sakei/fuchuensis*,* L. sakei/curvatus/graminis*, and *W. confusa*/*cibaria* (Figure 4c). The second phase (backslopping steps 5–7) showed the presence of *L. brevis, L. plantarum/brevis,* and *P. pentosaceus*. The last phase of the backslopping process (backslopping steps 8–10) was dominated by *L. brevis*.

Culture‐independent analysis based on 26S rRNA‐PCR‐DGGE fungal community profiling revealed the prevalence of *K. exigua* throughout the whole backslopped bakery teff sourdough production TF3′ (Figure 4f). No PCR amplicons could be generated for samples of TF1′and TF2′.

#### 3.3.2 Backslopped laboratory sourdough productions

Culture‐independent analysis based on 16S rRNA‐PCR‐DGGE bacterial community profiling revealed three different phases during the backslopped laboratory teff sourdough productions TF1 and TF2 (Figure 4d,e). The first phase (first two backslopping steps) of TF1 was dominated by *L. sakei*, followed by a second phase (backslopping steps 3–6) that was dominated by *L. fermentum* and *Le. citreum*. During the third phase of the backslopping process (backslopping steps 7–10), *L. fermentum* was the sole LAB species present. The first phase (backslopping steps 1–5) of TF2 was dominated by *W. cibaria/confusa*, although this LAB species was present till the end (Figure 4e). The second phase (backslopping steps 5–10) showed the dominance of *L. fermentum*.

Culture‐independent analysis based on 26S rRNA‐PCR‐DGGE fungal community profiling revealed the occurrence of *S. cerevisiae* throughout the whole backslopped laboratory teff sourdough production TF2 (Figure 4g). No PCR amplicons could be generated for samples of TF1.

#### 3.3.3 Starter culture‐initiated laboratory sourdough productions

**Figure 5:**
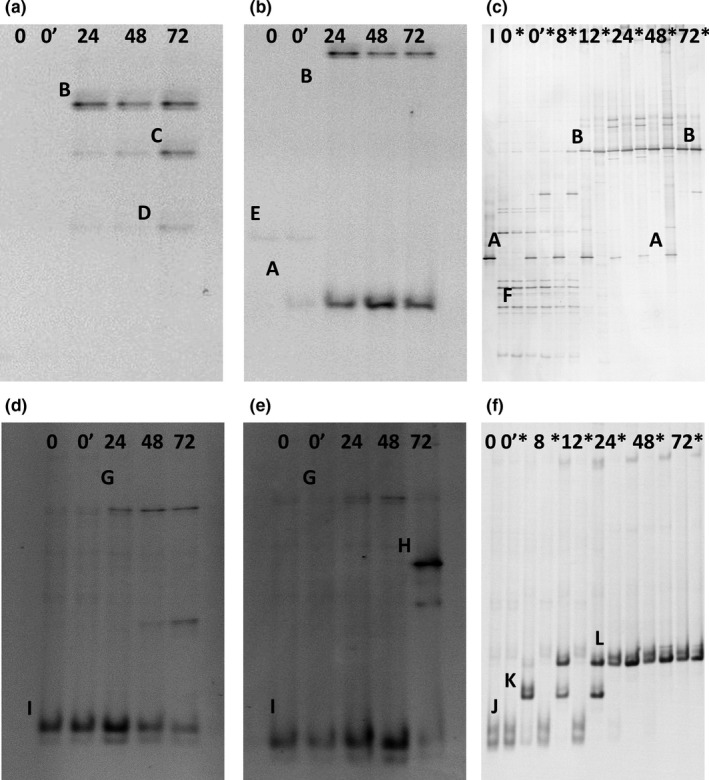
Culture‐independent bacterial (a–c) and fungal (d–f) community dynamics through 16S rRNA‐PCR‐ and 26S rRNA‐PCR‐DGGE (universal primers) analysis during *Lactobacillus sanfranciscensis* IMDO 150101‐initiated fermentor‐scale laboratory teff sourdough productions (a,d, TFFS1; b,e, TFFS2; c,f, TFFS3); I represents the inoculum, 0 represents the initial flour‐water mixture, 0′ represents the sourdough sample after addition of the starter culture strain; 8, 12, 24, 48, and 72 represent the sourdough samples taken after 8, 12, 24, 48, and 72 hr of fermentation, respectively. Plate washes are represented by an asterisk at the right of the respective time points. The bands were identified as: A, *Lactobacillus sanfranciscensis* (100% identity, accession no. NR_117814.1); B, *Weissella cibaria/confusa* (100% identity; accession no. LC096236.1/LC063164.1); C, *Lactobacillus fermentum* (99% identity, accession no. LC065036.1); D, *Acinetobacter oleivorans*/*pittii*/*baumannii* (100% identity, accession no. NR_102814.1/NR_117621.1/NR_117677.1); E, chloroplast DNA (98% identity, accession no. LC005978.1); F, *Microbacteriaceae* (100% identity, accession no. LC094672.1); G, no identification; H, *Cyberlindnera fabianii* (100% identity, accession no. EF550321.1); I, plant DNA (*Poaceae* spp., 95% identity; accession no. AK427333.1); J, fungal DNA (*Dothideomycetes*, 100% identity, accession no. KP330452.1); K, *Fusarium sublunatum*/*equiseti*/*chlamydosporum* (100% identity, accession no. KM231680.1/GQ505688.1/AY213706.1); and L, *Saccharomyces cerevisiae* (100% identity, accession no. NG_042623.1)

16S rRNA‐PCR‐DGGE bacterial community profiling revealed that *L. sanfranciscensis* IMDO 150101 as added starter culture strain did not prevail during two of the three teff fermentor‐scale sourdough fermentations carried out (Figure 5a–c). It did prevail during TFFS2, in combination with *W. cibaria*/*confusa*. The latter species was found in most fermentations, including TFSC30 and TFSC37.

Concerning the 26S rRNA‐PCR‐DGGE fungal community profiling, *S. cerevisiae* was solely found during TFFS3 (Figure 5d–f). Initially, other fungal species were retrieved as well, in particular species of plant pathogens. In the case of TFFS1 and TFFS2, one band of PCR amplicons could not be identified (Figure 5d,e). No other yeast species could be identified during TFFS1, whereas *Cyberlindnera fabianii* was detected during TFFS2. In all cases, plant DNA was found too.

### 3.4 Metabolite target analysis

#### 3.4.1 Backslopped bakery sourdough productions

**Figure 6:**
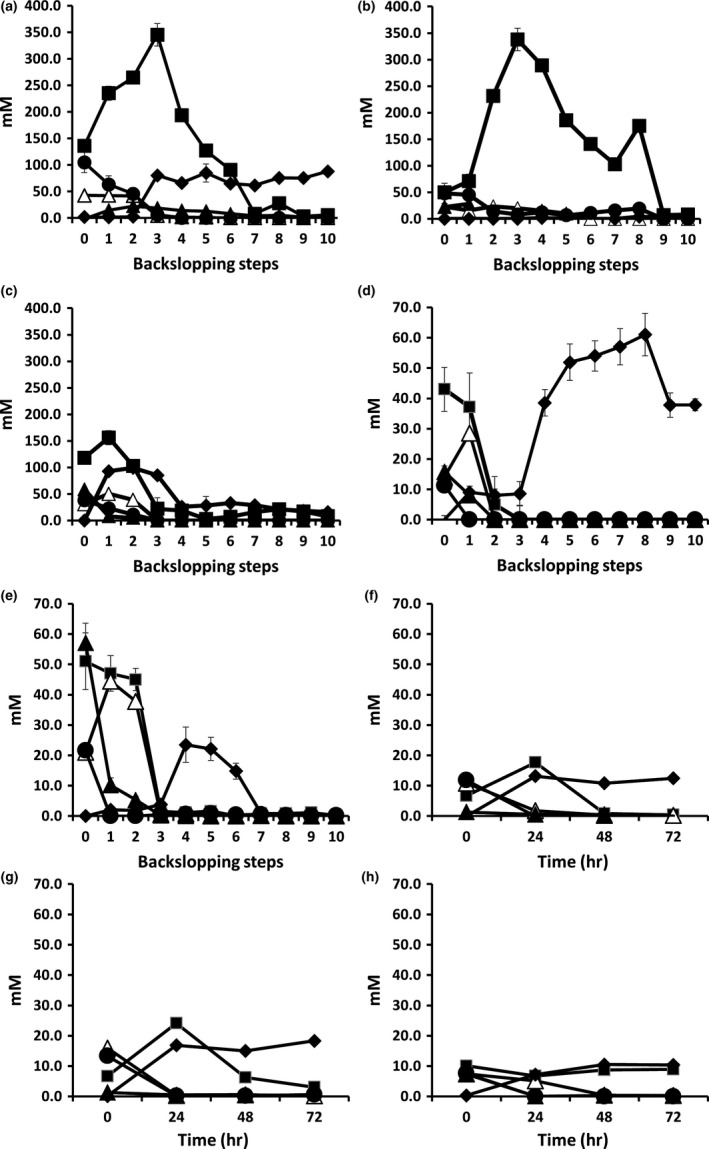
Evolution of the residual concentrations of maltose (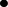), sucrose (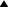), glucose (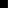), and fructose (△) and concentrations of mannitol (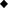) produced during teff sourdough productions carried out under bakery conditions (a, TF1′; b, TF2′, c, TF3′) and laboratory conditions (d, TF1; e, TF2) and during *Lactobacillus sanfranciscensis* IMDO 150101‐initiated fermentor‐scale laboratory teff sourdough productions (f, TFFS1; g, TFFS2; h, TFFS3)

**Figure 7:**
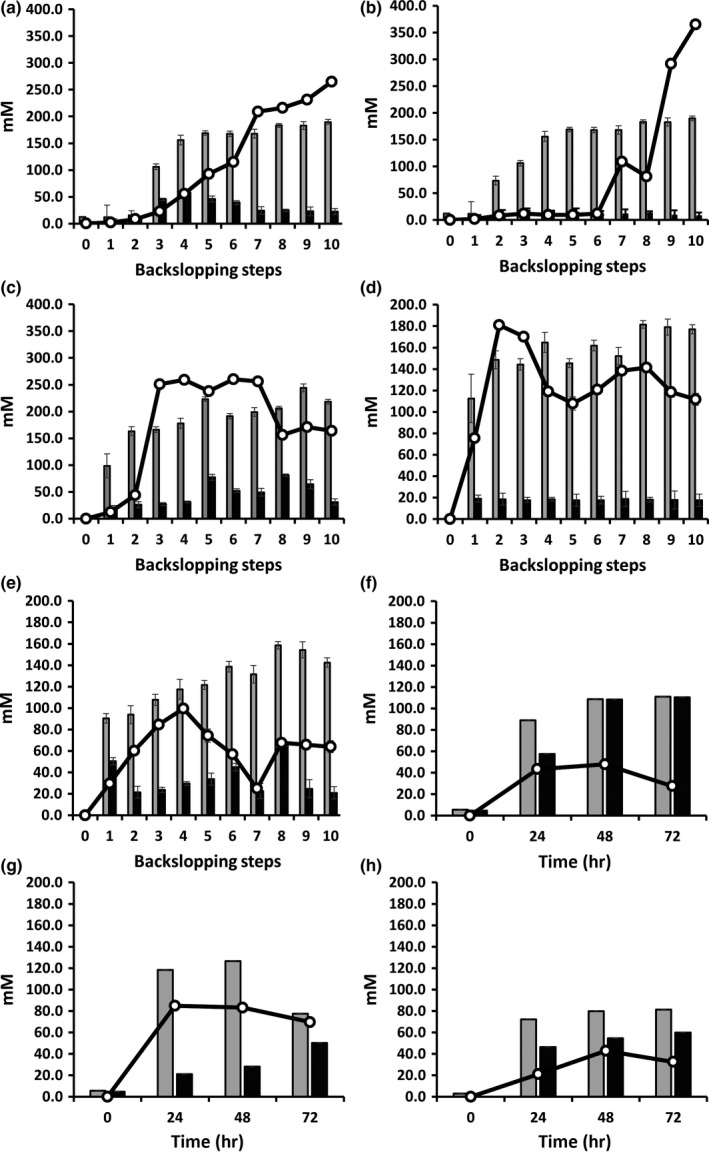
Evolution of the concentrations of lactic acid (gray bars), acetic acid (black bars), and ethanol (black lines) produced during spontaneous teff sourdough productions carried out under bakery conditions (a, TF1′; b, TF2′; c, TF3′) and laboratory conditions (d, TF1; e, TF2) and during *Lactobacillus sanfranciscensis* IMDO 150101‐initiated fermentor‐scale laboratory teff sourdough productions (f, TFFS1; g, TFFS2, h; TFFS3)

The unfermented dough mixtures of the three backslopped bakery teff sourdough productions TF1′, TF2′, and TF3′ consisted of 104.6 ± 19.0 mM, 48.2 ± 4.1 mM, and 38.6 ± 3.7 mM maltose, 135.8 ± 2.7 mM, 50.1 ± 5.8 mM, and 118.2 ± 5.7 mM glucose, 14.6 ± 2.2 mM, 22.8 ± 4.1 mM, and 59.2 ± 7.8 mM sucrose, and 42.8 ± 1.6 mM, 23.3 ± 1.6 mM, and 1.9 ± 2.8 mM fructose, respectively (Figure 6a–c). All carbohydrates were depleted toward the end of all bakery teff sourdough productions. During both TF1′ and TF3′, the concentrations of mannitol increased slowly to reach concentrations of 87.9 ± 1.4 mM and 46.0 ± 0.2 mM, respectively. Mannitol concentrations <5 mM were found during TF2′. Lactic acid was the main metabolite produced, and its concentrations increased during the third, second, and first backslopping steps of TF1′, TF2′, and TF3′, respectively, to reach concentrations of 189.7 ± 9.2 mM, 171.3 ± 4.8 mM, and 218.0 ± 4.4 mM at the end of the backslopping processes (Figure 7a–c). The second main metabolite produced was ethanol, the concentrations of which increased slowly during TF1′ and were delayed until backslopping step 6, followed by a fast increase, during TF2′, to reach concentrations of 265.0 ± 6.7 mM and 365.4 ± 2.3 mM at the end of the backslopping processes, respectively. The ethanol concentration produced during TF3′ reached its plateau after the third backslopping step (on average 252.8 ± 3.0 mM), followed by a decrease to 164.4 ± 5.6 mM at the end of the backslopping process. The concentrations of acetic acid reached a maximum of 57.8 ± 3.5 mM after the third backslopping step of TF1′, an average of 11.8 ± 2.2 mM during TF2′, and an average of 59.0 ± 5.4 mM during backslopping steps 5–10 of TF3′.

#### 3.4.2 Backslopped laboratory sourdough productions

The unfermented dough mixtures of both backslopped laboratory teff sourdough productions TF1 and TF2 were composed of 11.3 ± 2.1 mM and 21.6 ± 3.6 mM maltose, 43.8 ± 7.3 mM and 51.4 ± 9.4 mM glucose, 15.8 ± 4.5 mM and 57.1 ± 6.1 mM sucrose, and 14.1 ± 3.6 mM and 21.2 ± 3.9 mM fructose, respectively (Figure 6d,e). After the third backslopping step, all carbohydrates were depleted during both the TF1 and TF2 sourdough productions. Mannitol was produced from the beginning of both backslopping processes and reached maximal concentrations of 53.9 ± 4.1 mM and 23.5 ± 5.3 mM after the eight and fourth backslopping steps, respectively, followed by a decrease toward 37.9 and 0 mM at the end of both sourdough productions. Lactic acid was the main metabolite produced during both TF1 and TF2; its concentration increased steadily to reach maximal concentrations of 177.8 ± 4.3 mM and 142.5 ± 4.4 mM at the end of the respective backslopping processes (Figure 7d,e). The second main metabolite produced was ethanol, which increased until the second and fourth backslopping steps during TF1 (170.2 ± 3.1 mM) and TF2 (99.6 ± 1.6 mM), respectively, followed by a decrease upon further backslopping, to reach concentrations of 111.6 ± 2.1 mM and 63.9 ± 1.1 mM, respectively, at the end of the backslopping processes. Acetic acid was produced in low and moderate concentrations during TF1 and TF2, respectively, with maxima of on average 18.5 mM and 33.8 mM during the whole backslopping processes.

#### 3.4.3 Starter culture‐initiated laboratory sourdough productions

The unfermented dough mixtures of the three starter culture‐initiated fermentor‐scale teff sourdough fermentations TFFS1, TFFS2, and TFFS3 consisted of 12.0 ± 2.0 mM, 13.0 ± 1.0 mM, and 7.5 ± 0.1 mM maltose, 14.0 ± 3.0 mM, 10.0 ± 2.0 mM, and 13.0 ± 1.0 mM glucose, 14.0 ± 2.0 mM, 0.9 ± 0.1 mM, and 7.3 ± 1.2 mM sucrose, and 10.8 ± 0.2 mM, 16.0 ± 3.3 mM, and 7.4 ± 0.2 mM fructose, respectively (Figure 6f–h). Maltose and sucrose were quickly depleted (within 12 hr of fermentation). Glucose remained present throughout the entire 72‐hr fermentation course in the case of TFFS2 and TFFS3, while it was exhausted in the case of TFFS1. Fructose could not be found anymore after 48 hr of fermentation. Mannitol concentrations varied between 9.4 ± 0.7 (TFFS3) and 18.3 ± 0.7 mM (TFFS2). Lactic acid was the main metabolite found after 72 hr of fermentation, with concentrations of 111.0 ± 0.5 mM, 77.6 ± 0.1 mM, and 81.4 ± 1.7 mM, respectively (Figure 7f–h). The second main metabolite produced was acetic acid, whose concentrations were 110.5 ± 13.7 mM, 50.1 ± 0.9 mM, and 59.8 ± 10.7 mM, respectively. The concentrations of ethanol decreased toward the end of the 72‐hr fermentation period, and final concentrations of 27.0 ± 0.7 mM, 69.7 ± 2.4 mM, and 32.5 ± 0.8 mM were found, respectively.

#### 3.4.4 Volatile compounds

**Figure 8:**
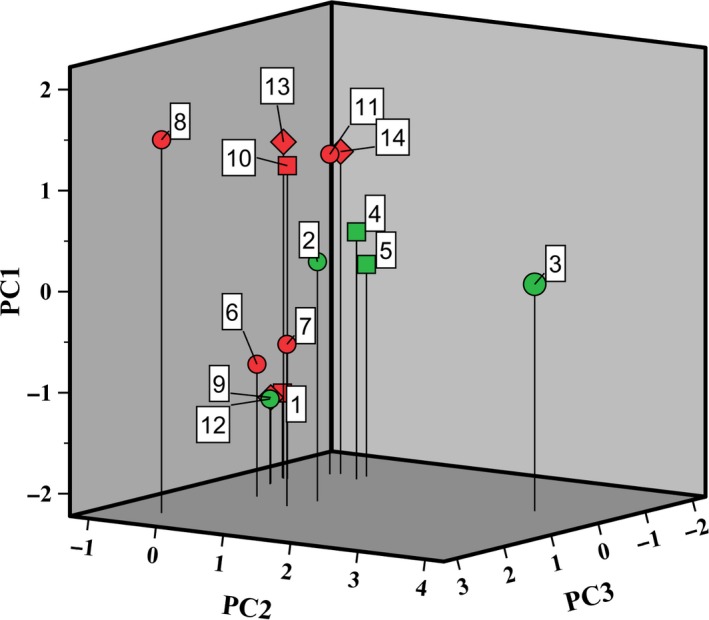
Principal component analysis of the HS/SPME‐GC‐MS volatile analysis data (peak areas) of the backslopped teff sourdough productions under bakery conditions [red; TF1′: 6, unfermented; 7, after five backslopping steps; 8, after ten backslopping steps; TF2′: 9, unfermented; 10, after five backslopping steps; 11, after ten backslopping steps; and TF3′: 12, unfermented; 13, after five backslopping steps; 14, after ten backslopping steps] and laboratory conditions [green; TF1: 1, unfermented; 2, after five backslopping steps; 3, after ten backslopping steps; and TF2: 4, after five backslopping steps; 5, after ten backslopping steps]

A total of 128 volatile compounds was identified in samples from the backslopped teff sourdough productions through HS/SPME‐GC‐MS, including eight carboxylic acids, 30 alcohols, 19 aldehydes, five alkenes, one dioxolane, 22 esters, six furans, seven other hydrocarbons, 18 ketones, six phenols, four pyrazines, and one terpene. The peak area data of the HS/SPME‐GC‐MS volatile analysis were used to perform a statistical analysis. A PCA revealed three principal components (PCs) accounting for 74% of the total variance of these data. PC1 (36.6% of the total variance) was characterized by strong negative factor loadings for aldehydes and ketones, and strong positive factor loadings for alcohols, esters, and carboxylic acids. PC2 (21.3% of the total variance) was characterized by strong positive factor loadings for miscellaneous compounds, comprising hydrocarbons. PC3 (15.8% of the total variance) was characterized by strong positive factor loadings for C8‐C10 esters. A three‐dimensional score plot (Figure 8) of the resulting data revealed the formation of specific volatile compounds during backslopped teff sourdough productions. All unfermented sourdough samples were negatively correlated with PC1, independent of the process conditions (laboratory or bakery). The bakery teff sourdough samples of backslopping steps 10 and 5 of TF1′ and TF3′, respectively, were positively correlated with PC1. The last sample of TF1 was positively correlated with PC2. The laboratory and bakery teff sourdough samples of backslopping steps 10 of TF2 and TF3′, respectively, were negatively correlated with PC3. The bakery teff sourdough samples of backslopping steps 5 and 10 of TF1′ were positively correlated with PC3. In general, the differences between the backslopped laboratory and bakery sourdough productions indicated that the former were characterized by the abundance of phenols, aldehydes, and furans (and hydrocarbons in mature sourdough of TF1), whereas the latter were characterized by the absence of aldehydes and ketones and the presence of alcohols, esters, and acids.

#### 3.4.5 Bread production and evaluation

Based on the descriptive results from the 21 consumers involved, the organoleptic properties of the wheat‐based breads made with teff sourdoughs showed differences compared with the all‐wheat‐based reference breads. The breads made with mature laboratory and bakery teff sourdoughs showed a decrease in volume compared to the reference breads. Independently of the sourdoughs used, the teff sourdough‐based breads showed a darker crust and crumb and possessed an acid, grain‐like, and malty crumb taste. The sourdough breads made with the bakery teff sourdoughs further possessed distinct flavor compounds.

## 4 DISCUSSION

The use of teff flour and teff sourdough is very limited; its most known application is to prepare *injera* in Ethiopia and Eritrea (Ashenafi, 2006; Gebremariam et al., 2014). However, given its high‐quality protein, slowly digestible complex carbohydrates, many fibers, and bioavailable minerals, it is very promising for use in human food production, in particular for bread production (Campo et al., 2016).

In the present study, teff sourdough productions were performed through backslopping under different process conditions in different environments (spontaneous backslopped fermentations in laboratory and bakery settings and flour‐native starter culture‐initiated fermentations in the laboratory). The backslopped teff sourdoughs were characterized by the presence of the LAB species *L. fermentum*,* L. plantarum, L. sanfranciscensis*, and *W. cibaria* and the yeast species *S. cerevisiae* in the case of the laboratory productions (high temperature, high dough yield, moderate pH decrease, pH < 4.0 after five backslopping steps) and of the LAB species *L. brevis, L. helveticus, L. plantarum, L. sanfranciscensis*, and *P. pentosaceus* and the yeast species *K. exigua* in the case of the bakery productions (low temperature, low dough yield, moderate pH decrease, pH < 4.0 after five backslopping steps), as assessed by the culture‐dependent and ‐independent methods applied. Whereas the backslopped laboratory teff sourdough production TF1 was at its mature stage dominated by *L. fermentum* in the presence of *L. sanfranciscensis,* the backslopped laboratory teff sourdough production TF2 was dominated by *W. cibaria* throughout the whole backslopping period. This reflects differences in the microbial composition of the teff flour and processing conditions such as temperature and pH evolution (Van Kerrebroeck et al., 2016; Vrancken et al., 2011). During the *L. sanfranciscensis* IMDO 150101‐initiated teff sourdough fermentations, the added starter culture strain only prevailed at 23°C (solely) and in certain fermentations carried out at 30°C (in combination with *L. fermentum*, pediococci, and weissellas). Almost all LAB species mentioned above were retrieved from the teff flour too. Although dominance of leuconostocs and weissellas is considered typical for high‐pH sourdoughs, they have been found in low‐pH sourdoughs too (Settanni et al., 2013; Valmorri et al., 2006). Furthermore, bakery sourdoughs can also contain acid‐tolerant lactobacilli (Scheirlinck et al., 2008). In contrast, the backslopped bakery sourdough productions TF2′ and TF3′ were dominated by *L. helveticus* and *L. brevis*, respectively. *Lactobacillus helveticus* has been selected for during sorghum and rye sourdoughs before (Hamad, Dieng, Ehrmann, & Vogel, 1997; Viiard, Mihhalevski, Ruhka, Paalme, & Sarand, 2013). During these fermentations, it showed a high adaption to the specific flour used and fermentation conditions applied (Viiard et al., 2013). All the differences found during the present study reflect the course of the respective fermentation processes that are determined by the properties of the flour batch and the environmental and processing conditions and thus underline the variability of spontaneous sourdough fermentation processes, especially when performed under not completely controllable circumstances, as is the case in small industrial or artisan bakeries.

The properties of the teff flours were of great influence. Presumably, the variations in composition of the three teff flours used contributed to the differences found in the evolution of pH and TTA, in particular during the first backslopping step of the backslopped teff sourdough productions. High TTA values reflect high concentrations of lactic acid and acetic acid, thanks to a high buffer capacity of teff flour due to its high ash content, and hence the dominance of mainly heterofermentative LAB species; in turn, they imply high acid tolerance of the dominating LAB and yeast species (Van Kerrebroeck et al., 2016). Furthermore, batch variations in dominating LAB and yeast species are common during sourdough productions and reflect not only the impact of isolation and identification procedures but also the quality of the flour in terms of age, microbial load, stability, *etc*., as well as environmental contamination (De Vuyst et al., 2014, 2017; Huys, Daniel, & De Vuyst, 2013; Minervini et al., 2015). For instance, under the laboratory conditions applied in the study of Moroni et al. (2011), no common LAB species were found in two types of sourdough produced from the same flour either. One type of teff sourdoughs was dominated by two LAB species only, that is, *P. pentosaceus* and *Le. holzapfelii*, and the yeast species *K. barnettii*. A second type of teff sourdoughs harbored several lactobacilli and the yeast species *C. glabrata*. However, unique isolations such as *Le. holzapfelii* may be accidental occurrences (De Vuyst et al., 2014, 2017). Furthermore, given the production conditions of teff, batch‐to‐batch variations are possibly larger than with commercial wheat flour (Gebremariam et al., 2014). Teff kernels are milled as a whole and, hence, a larger and possibly more variable microbial contamination can be expected (Berghofer, Hocking, Miskelly, & Jansson, 2003; Gebremariam et al., 2014). This was reflected in the presence of plant pathogens at the start of certain of the teff sourdough fermentations performed.

Most of the LAB species found in the mature teff sourdoughs of the present study have been isolated from sourdoughs before (De Vuyst et al., 2014, 2017), including teff sourdoughs (Desiye & Abegaz, 2013; Moroni et al., 2010, 2011). The presence of strictly heterofermentative *L. fermentum* (fermenter of maltose and producer of lactic acid and acetic acid as well as mannitol, the latter by the use of fructose as alternative external electron acceptor) and facultatively heterofermentative *L. plantarum* (fermenter of maltose, glucose, and fructose and producer of lactic acid), accommodates the LAB species diversity of several backslopped sourdough productions (wheat, rye, spelt, and barley) under laboratory conditions (Van der Meulen et al., 2007; Vrancken et al., 2011; Weckx, Van der Meulen, Allemeersch et al., 2010; Weckx, Van der Meulen, Maes et al., 2010; Weckx et al., 2011). These LAB species dominate after a three‐step evolution of the microbial communities of the flour, being a succession of nonacid‐tolerant LAB species, sourdough nonspecific LAB species, and acid‐tolerant sourdough‐specific LAB species upon backslopping. *Lactobacillus pontis* was not retrieved from the teff sourdoughs produced during the present study, although this LAB species was considered to be able to dominate teff sourdough fermentations (Moroni et al., 2010, 2011). It is however not commonly present in spontaneous sourdough fermentations, probably because of its limited carbohydrate utilization pattern (Vogel et al., 1994). *Lactobacillus brevis*, considered to be able to dominate teff sourdough fermentations as well (Moroni et al., 2010), did occur in some backslopped bakery teff sourdoughs of the present study. Its presence may reflect the lower ambient temperature and higher carbohydrate content, in particular fructose, of the backslopped bakery sourdoughs (De Vuyst et al., 2002). Furthermore, the high glucose concentrations, owing to the lack of β‐amylase activity in teff flour, could have contributed to the occurrence of *Weissella* species, as these species preferably grow on glucose (Galle, Schwab, Arendt, & Gänzle, 2010; Gänzle, 2014; Gebremariam, Zarnkow, & Becker, 2013; Gebremariam et al., 2014; Moroni et al., 2011). Additionally, degradation of phenolic compounds has been demonstrated for *L. brevis*, pediococci, and weissellas (Filannino, Gobbetti, De Angelis, & Di Cagno, 2014). The yeast species *K. exigua* and *S. cerevisiae* are commonly associated with sourdough fermentations (Lhomme et al., 2016; Vrancken et al., 2010). Whereas *K. exigua* (maltose‐negative) is part of a mutualistic interaction with *L. sanfranciscensis* and/or *L. brevis* (both maltose‐positive) in stable sourdoughs, this is not considered to be the case for *S. cerevisiae* (Gobbetti, 1998; Kline & Sugihara, 1971; Lhomme et al., 2016; Ottogalli, Galli, & Foschino, 1996). Other yeast species usually dominate in the absence of *S. cerevisiae* or *Kazachstania* spp. (Moroni et al., 2011; Vrancken et al., 2010). Based on all these and former data, it can be concluded that *L. fermentum* is an interesting starter culture for (teff) sourdough fermentations performed at high temperature (≥30°C) and high dough yield, whereas *L. brevis*, pediococci, and weissellas are interesting starter cultures for fermentations at ambient temperature and low dough yield.

As *L. sanfranciscensis* was present under both laboratory and bakery conditions in the backslopped teff sourdoughs of the present study, it could be a valuable alternative as starter culture for use in teff sourdough fermentations performed at low temperature, for example in bakery settings. Indeed, the present study showed that sourdough fermentations based on teff flour allowed the growth of *L. sanfranciscensis*, both in the laboratory and in a small industrial bakery, if the conditions were suitable for its prevalence, in particular with respect to temperature (better at 23°C than at 30°C) and dough yield (preferring firm above liquid sourdoughs due to the higher pH of the former) (De Vuyst et al., 2014; Viiard et al., 2016; Vogelmann & Hertel, 2011). Production conditions in bakery settings are characterized by a lower temperature, lower dough yield, and slower acidification of the cereal matrix in comparison with laboratory fermentation conditions. However, an appropriate fermentation temperature in connection with slow acidification allowed *L. sanfranciscensis* to grow during certain laboratory teff fermentations. In the past, the growth of *L. sanfranciscensis* was associated with wheat sourdoughs produced under certain conditions of temperature and pH (Hammes et al., 2005), explaining its wide occurrence in Chinese, French, Greek, Italian, and San Francisco wheat sourdoughs (De Vuyst et al., 2002; Kitahara, Sakata, & Benno, 2005; Kline & Sugihara, 1971; Lattanzi et al., 2013; Lhomme, Orain, Courcoux, Onno, & Dousset, 2015; Lhomme et al., 2015; Liu et al., 2016; Minervini et al., 2012; Zhang et al., 2015). Yet, it has been isolated from rye sourdoughs (Kitahara et al., 2005; Spicher, 1984; Spicher & Lönner, 1985; Spicher & Schröder, 1978) and spelt sourdoughs (Scheirlinck et al., 2007, 2008) too. However, *L. sanfranciscensis* could not be retrieved from diverse wheat, rye, spelt, and barley sourdoughs backslopped in the laboratory (Harth et al., 2016; Van der Meulen et al., 2007; Vrancken et al., 2011; Weckx, Van der Meulen, Allemeersch et al., 2010; Weckx, Van der Meulen, Maes et al., 2010; Weckx et al., 2011) neither was it competitive in backslopped sorghum sourdoughs (Sekwati‐Monang, Valcheva, & Gänzle, 2012). The fermentation temperature and dough yields applied were probably responsible for this. Further, it has been speculated that the presence of phenolic compounds in the flour from grains of C4‐plants, such as teff and sorghum, inhibits the growth of *L. sanfranciscensis* (Gänzle, 2014; Sekwati‐Monang et al., 2012). Yet, the retrieval of *L. sanfranciscensis* from spontaneous teff sourdoughs and its prevalence in certain laboratory teff sourdoughs during the present study underlines that its selection is rather steered by technological process parameters, such as temperature and pH, as well as its preference for maltose as energy source and its association with maltose‐negative yeasts, such as *Candida humilis* (now reclassified as *Kazachstania humilis*) and *K. exigua* (De Vuyst et al., 2014, 2017). Its presence in the flour and the occurrence of the right environmental and processing conditions for its growth will hence determine its prevalence in sourdoughs spontaneously developed. However, the microbial load of the flour will depend on agricultural crop practices (use of manure), weather conditions (sunshine and rainfall influencing mold growth and hence causing microbial competition), and its contact with rodents and insects throughout the chain from cereal to bread (Boiocchi et al., 2017; Groenewald, Van Reenen, & Dicks, 2006; Minervini et al., 2015; Thaochan, Drew, Chinajariyawong, Sunpapao, & Pornsuriya, 2015). Given the milling of the small teff seeds as a whole and their high surface/volume ratio (Gebremariam et al., 2014; Tefera & Belay, 2006), a high microbial load together with a variable microbial species diversity can be expected in the case of teff flour. The recovery of *L. sanfranciscensis* from insect frass indicates a probable contamination route of teff flour with *L. sanfranciscensis*, a species that is typical for sourdoughs but rarely recovered elsewhere (Boiocchi et al., 2017; De Vuyst et al., 2017). Alternatively, berries and flowers might be the carrier for *L. sanfranciscensis* (Ripari, Gänzle, & Berardi, 2016).

The teff sourdoughs of the present study were rich in volatile compounds and had an impact on the flavor of the breads produced thereof. In general, sourdoughs harbor a wide spectrum of volatile compounds, which originate from enzymatic and chemical reactions involving flour substrates (e.g., aldehydes and alcohols from lipid oxidation) and both bacterial and yeast metabolism (e.g., alcohols, aldehydes, ketones, carboxylic acids, and esters from enzymatic conversions) (Gänzle, 2014). The differences found between sourdoughs produced under laboratory and bakery fermentation conditions only partially aligned with the differences found between liquid and firm sourdoughs (Di Cagno et al., 2014), possibly due to the different process conditions applied during the sourdough productions presented here and the differences in microbial species prevailing.

## 5 CONCLUSIONS

Backslopped teff sourdoughs are characterized by different microbial species, based on the process conditions applied during their production. This was illustrated by the prevalence of different LAB species in bakery and laboratory teff sourdoughs produced under different environmental conditions, and the prevalence of a *L. sanfranciscensis* strain in bakery and laboratory teff sourdoughs fermented at low temperature (<30°C). In general, the use of representative strains of *L. fermentum*,* L. brevis*,* L. sanfranciscensis*, pediococci, and weissellas, adapted to the environmental conditions they will be confronted with, will contribute to the production of stable teff sourdoughs and flavorful baked goods produced thereof.

## CONFLICT OF INTEREST

None declared.

## ETHICAL STATEMENT

This study did not involve human or animal testing. Consumers (21) were involved to assess the breads produced.
